# Hydroxyapatite Whisker Reinforced 63s Glass Scaffolds for Bone Tissue Engineering

**DOI:** 10.1155/2015/379294

**Published:** 2015-03-04

**Authors:** Cijun Shuai, Yiyuan Cao, Chengde Gao, Pei Feng, Tao Xiao, Shuping Peng

**Affiliations:** ^1^State Key Laboratory of High Performance Complex Manufacturing, Central South University, Changsha 410083, China; ^2^Orthopedic Biomedical Materials Institute, Central South University, Changsha 410001, China; ^3^Department of Orthopedics, The Second Xiangya Hospital, Central South University, Changsha 410001, China; ^4^Hunan Provincial Tumor Hospital and the Affiliated Tumor Hospital of Xiangya School of Medicine, Central South University, Changsha 410006, China; ^5^School of Basic Medical Science, Central South University, Changsha 410078, China

## Abstract

Bioactive glass (BG) is widely used for bone tissue engineering. However, poor mechanical properties are the major shortcomings. In the study, hydroxyapatite nanowhisker (HANw) was used as a reinforcement to improve the mechanical properties. 63s glass/HANw scaffolds were successfully fabricated by selective laser sintering (SLS). It was found that the optimal compressive strength and fracture toughness were achieved when 10 wt.% HANw was added. This led to 36% increase in compressive strength and 83% increase in fracture toughness, respectively, compared with pure 63s glass scaffolds. Different reinforcement mechanisms were analyzed based on the microstructure investigation. Whisker bridging and whisker pulling-out were efficient in absorbing crack propagating energy, resulting in the improvement of the mechanical properties. Moreover, bioactivity and biocompatibility of the scaffolds were evaluated in vitro. The results showed that composite scaffolds with 10 wt.% HANw exhibited good apatite-forming ability and cellular affinity.

## 1. Introduction

BG of the SiO_2_–CaO–P_2_O_5_ system has attracted increasing attention as a promising bone scaffold material [1–3]. It can convert to hydroxyl-carbonate apatite (HCA) similar to the main mineral constituent of nature bone and bond firmly with surrounding tissues [4, 5]. Calcium ions and phosphate ions, which are released from BG, can further promote osteogenesis and activate osteogenic gene expression [6]. In addition, recent studies have indicated that BG can also induce neovascularization, enhancing the body's self-rehabilitation capacity [7]. 63s glass, a new generation of BG with molar composition of 63% SiO_2_, 28% CaO, and 9% P_2_O_5_, has excellent bioactive and resorbent properties [8–10]. However, the poor mechanical properties have prevented it from further applications. Therefore, a considerable effort has been made to improve mechanical properties. Generally, reinforcement by ceramic particles or whiskers has been considered an effective way [11].

Hydroxyapatite (HA) is one of the most biocompatible ceramic materials which has been studied extensively and clinically used due to the good bioactivity, high osteoconductive, and excellent osteoblastic responses [12–14]. HA has similar mineral constituents to nature bones and can directly bond to the bone [15]. In addition, the latest work has shown that HANw and HA nanoparticle (HANp) were helpful in improving mechanical properties of polymers and calcium phosphate ceramics. Converse et al. investigated the effects of HANw reinforcement on mechanical properties of polyetheretherketone (PEEK) and found that elastic modulus and tensile strength could increase effectively [16]. Hong et al. added HANp into PLLA composite and found that tensile strength and bending strength had noticeable improvement [17]. Hu et al. studied porous *β*-TCP scaffolds reinforced with HANw [18]. The results showed that HANw not only improved the mechanical properties but also enhanced bioactivity of the scaffolds.

In the present study, 63s glass/HANw scaffolds were manufactured by using SLS. For comparison, HANp added 63s glass scaffolds had been also prepared. The influence of HANw on the microstructure and mechanical properties of 63s glass scaffolds were investigated. The reinforcement mechanisms were also analyzed. In addition, bioactivity and biocompatibility of the scaffolds were evaluated in vitro.

## 2. Materials and Methods

### 2.1. Starting Materials

63s glass (prepared by the sol-gel method) with a nominal chemical composition of 63% SiO_2_, 28% CaO, and 9% P_2_O_5_ in molar percentages was purchased from the KunShan Chinese Technology New Materials Co. Ltd. It is irregular in shape with an average size of 0.2–10 *μ*m. Two different kinds of HA (nanowhisker and nanoparticle) were provided by Nanjing Emperor Nano Material Co. Ltd (China). The length and aspect ratio of the HANw are approximately 500 and 50 nm, respectively. The average size of the HANp is 100 nm.

### 2.2. Preparation of Composites

63s glass/HANw composites with HANw contents of 0, 5, 10, 15 and 20 wt.% were prepared using the following procedure: HANw was dispersed in ethanol and sonicated for 1 h. Then 63s glass powder, which was weighed in different amounts, was added to the HANw suspension and ball-milled for 12 h using ZrO_2_ balls as the grinding media. After mixing, the prepared mixture was dried at 353 K in an infrared stove to remove the liquid phases. Similarly, 63s glass/HANp composites with different weight ratio of 95/5, 90/10, 85/15, and 80/20 were also prepared.

### 2.3. Preparation of Scaffolds

**Figure 1:**
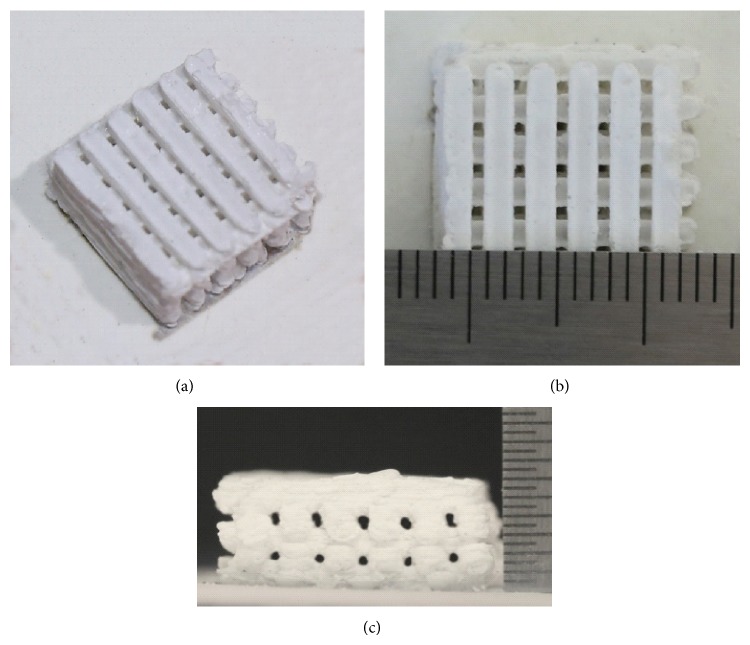
The porous 63s glass scaffolds with 10 wt.% HANw (a) isometric view; (b) top view; (c) side view.

A self-developed selective laser sintering system, as reported previously [19, 20], was used to prepare 3D porous composite scaffolds. It is made up of a CO_2_ laser with a focus system, a three-dimensional motion platform, and a corresponding control system. The CO_2_ laser, with a maximum output power of 100 W, was purchased from SYNRAD Co. in USA. The minimum laser spot diameter can reach 100 *μ*m with the focus system. The 3D motion platform is driven by servomotor for precise positioning. The control system is available to determine the movements of 3D motion platform and the laser output power. Optimal SLS process parameters were chosen: laser power of 7.0 W, laser spot diameter of 1.2 mm, scan speed of 100.0 mm/min, and layer thickness of about 0.2 mm. Then the porous scaffolds of uniform size (15 × 15 × 6 mm^3^) were fabricated. After removal of the unprocessed powder using compressed air, the interconnected porous structures were displayed in Figure 1. The porosity of the scaffolds was measured using the Archimedes method. The porosity can be calculated as 46.6 ± 2.5% by the following [21]:

(1) $$\begin{array}{c} P=\frac{\left(V_{a}-V_{t}\right)}{V_{a}}\times 100\%, \end{array}$$

where *V*_*a*_ is the apparent volume (mm^3^), *V*_*t*_ is the true volume (mm^3^), and *P* is the porosity (%).

### 2.4. Characterization

The morphology of the scaffolds was observed using SEM (TESCAN MIRA3 LMU, CO., Czech) equipped with an energy dispersive spectroscopy (EDS) instrument. The acceleration voltage applied was 20 kV. Before the SEM observations, the scaffolds were coated with platinum using a sputter coater (JFC-1600, JEOL CO., Japan). EDS analyses were performed to define the presence of HCA on the scaffolds surface after immersion in SBF. The functional group analyses were performed by FTIR with Nicolet 6700 spectrometer (Thermo Scientific Co. USA). The measurements were carried out in the mid-infrared range (400–4000 cm^−1^) at 0.6329 cm/s mirror speed. The phase compositions of the scaffolds were evaluated using XRD (D8-ADVANCE, Bruker AXS Inc., Germany) after ball-milling for 6 h. The data were recorded in the interval 10° ≤ 2*θ* ≤ 70° at the rate of 2°/min with Cu-K*α* radiation (1.54056 Å).

For the compressive strength tests, the 63s glass/HANw and 63s glass/HANw composites with a thickness of 1.2 mm were prepared to rectangular strips, ~1.3 mm width and 8 mm in length. The samples were loaded at a crosshead speed of 0.5 mm/min using a universal testing machine (Shanghai Zhuoji instruments Co. LTD, China). The fracture toughness was evaluated by indentation with a Vickers hardness tester (HXD-1000TM/LCD, Digital Micro Hardness Tester, Shanghai Taiming Optical Instrument Co. Ltd). The samples (8 × 1.3 × 1.2 mm^3^) were inlaid in epoxy resin, polished with sandpaper, and subjected to indentation on the surfaces. The average values of fracture toughness were calculated from five tests. The fracture toughness *K*_*ic*_ was determined using the following [22]:

(2) $$\begin{array}{c} K_{ic}=0.0824Pc^{-3/2}, \end{array}$$

where *P* is the indentation load (MN) and *c* is the radial crack length (m).

### 2.5. Bioactivity

The bioactivity of the 63s glass scaffolds with 10 wt.% HANw and 63s glass scaffolds with 10 wt.% HANp was evaluated by examining HCA formation in SBF which was prepared as previously proposed by Kokubo and Takadama [23] and had similar ion concentrations to those in human blood plasma. Scaffolds with thickness of 6 mm and dimensions of 15 × 15 mm^2^ were selected, and the ratio of solution volume to sample mass was kept constant at 1 mL·mg^−1^. The solutions with the samples were then kept in a shaking incubator at a controlled temperature of 36.5°C for 7 days. The SBF solutions were refreshed every 24 h. After the preselected immersing time, the scaffolds were removed from SBF, gently rinsed with distilled water, and then dried in vacuum desiccators. The surface deposits were examined by FTIR spectroscopy and SEM equipped with EDS microanalysis.

### 2.6. Cell Culture

The MG-63 cells derived from human osteosarcoma (ATCC, Rockville, MD) were used in this study to evaluate the biocompatibility of the 63s glass scaffolds with 10 wt.% HANw and 63s glass scaffolds with 10 wt.% HANp (15 × 15 × 6 mm^3^). The cells were seeded in 50 mL culture flask with fresh culture media supplemented with 10% fetal bovine serum (FBS, ATCC), 100 *μ*g/mL streptomycin, and 100 U/mL penicillin at 37°C in a humid atmosphere of 5% CO_2_. Before confluence, the cells were detached with trypsin/EDTA and resuspended in the medium. The scaffolds were sterilized by 75% ethanol and rinsed with phosphate-buffered saline (PBS). Then the cells at a concentration of 5 × 10^3^ cells/cm^2^ were added to the prewetted scaffolds.

### 2.7. Cell Morphology

Cells morphology on composite scaffolds was assessed by SEM observation. After 5 days of culture. Scaffolds were washed with PBS to eliminate unattached cells and fixed with 2.5% glutaraldehyde for 2 h. Following this, scaffolds of fixed cells were dehydrated in a series of graded ethanol (30, 50, 70, 95, and 100%) and further dried using hexamethyldisilazane (HMDS). Dried scaffolds were then sputter-coated with gold and examined using SEM.

## 3. Results and Discussion

### 3.1. Microstructure

**Figure 2:**
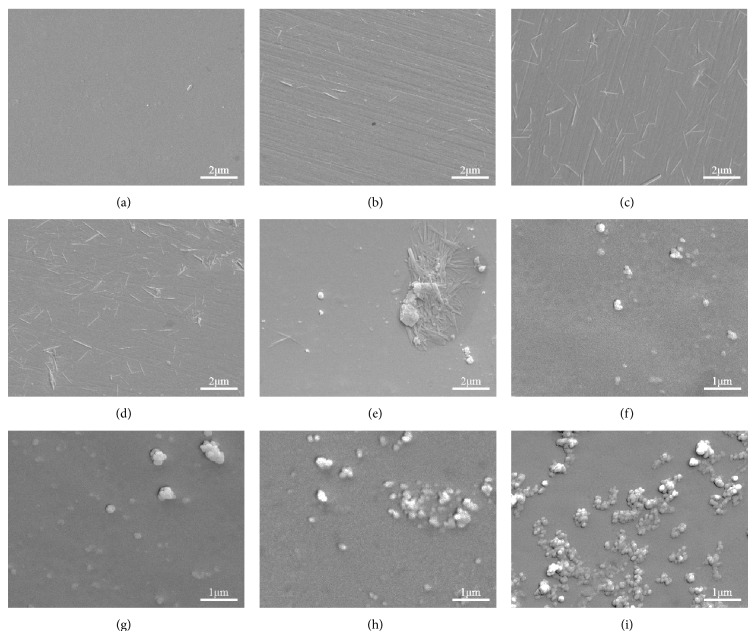
SEM micrograph of 63s glass/HANw scaffolds containing (a) 0 wt.%, (b) 5 wt.%, (c) 10 wt.%, (d) 15 wt.%, (e) 20 wt.% HANw and 63s glass/HANp scaffolds containing, (f) 5 wt.%, (g) 10 wt.%, (h) 15 wt.%, and (i) 20 wt.% HANp.

The microstructures of scaffolds were shown in Figure 2. A relatively smooth surface of 63s glass scaffolds was presented in Figure 2(a). When 10 wt.% HANw was added to the scaffolds, HANw was well-distributed (Figure 2(c)). With further increase in HANw content, some agglomerates of whisker could be observed (Figures 2(d) and 2(e)). In addition, the diameter of the HANw on the scaffolds was rougher than that of HANw before sintering. The fact showed that the HANw was coated with 63s glass, indicating strong interface physical bonding between amorphous 63s glass and HANw. On the other hand, the HANp was uniformly dispersed in the 63s glass matrix when the HANp content reached 5 wt.% (Figure 2(f)). As the HANp further increased, aggregates and loosely embedded HANp could be observed due to its high specific surface area, especially for highly filled composites.

**Figure 3:**
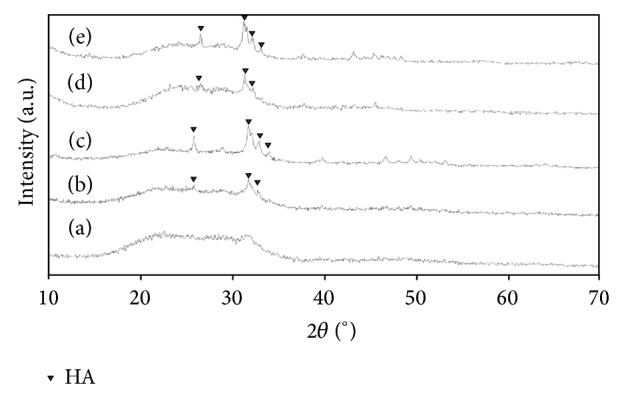
XRD patterns of (a) 63S glass scaffolds, 63s glass/HANw scaffolds with different HANw contents of (b) 5 wt.%, (c) 15 wt.%, 63s glass/HANp scaffolds with different HANp contents of (d) 5 wt.%, (e) 15 wt.%.

The microstructures of 63s glass scaffolds with and without reinforcement were further examined by using XRD (Figure 3). The results showed that no crystallization occurred in the XRD pattern of 63s glass scaffolds (Figure 3(a)), which indicated that the 63s glass maintained amorphous state. The XRD pattern of 63s glass scaffolds reinforced with HANw (Figures 3(b) and 3(c)) or with HANp (Figures 3(d) and 3(e)) both had common diffraction peaks at 2*θ* degree of 25.9° (002), 31.8° (211), 32.9° (300), and 34.1° (202) and was in good agreement with standard hydroxyapatite PDF Card (no. 9-432). No impurity phase was observed, which demonstrated that no reaction occurred between 63s glass and HA. In addition, the peaks intensity of HANw or HANp became stronger with the increasing content of HANw or HANp.

### 3.2. Mechanical Properties

**Figure 4:**
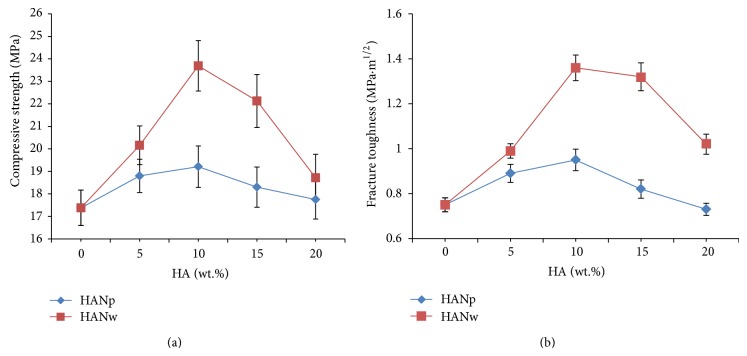
Mechanical properties of the 63s glass/HANw scaffolds and the 63s glass/HANp scaffolds: compressive strength and (b) fracture toughness.

The effect of HANw or HANp on the compressive strength and fracture toughness of the scaffolds was shown in Figure 4. The compressive strength increased linearly firstly and then decreased slightly (Figure 4(a)). The highest compressive strength of 63s glass/HANw scaffolds was 23.69 MPa, when the content of HANw increased to 10%, while it was 19.21 MPa for 63s glass/HANp scaffolds. This was about 36% increase in compressive strength for the scaffolds with 10 wt.% HANw addition compared with that of the pure 63s glass scaffolds. The decrease of compressive strength with addition of HANw or HANp more than 10 wt.% was attributed to the agglomeration of HANw or HANp.

The peak values of fracture toughness were found for HANw and HANp at 10 wt.% (Figure 4(b)). The HANw gave a significantly greater increase in fracture toughness than did the HANp. The maximum fracture toughness obtained from the 63s glass/HANw scaffolds was 1.36 MPa·m^1/2^ and that of 63s glass/HANp scaffolds was 0.95 MPa·m^1/2^. It suggested that HANw was more efficient in improving the mechanical properties of 63s glass scaffolds than HANp. The compressive strength of the 63s glass scaffolds with 10 wt.% HANw was higher than that of cancellous bone (0.1–16 MPa) [24]. The fracture toughness was a little lower than that of cortical bone (2–12 MPa·m^1/2^) [25]. Fu et al. applied a polymer foam replication technique to prepare the 13–93 bioactive glass scaffolds (porosity of 85 ± 2%, pore size of 100–500 *μ*m) [26]. The compressive strength of the obtained scaffolds was 11 ± 1 MPa. Yazdanpanah et al. used 30 wt.% of nanocrystalline forsterite to improve the fracture toughness of BG [27]. The fracture toughness was 0.22 MPa·m^1/2^.

**Figure 5:**
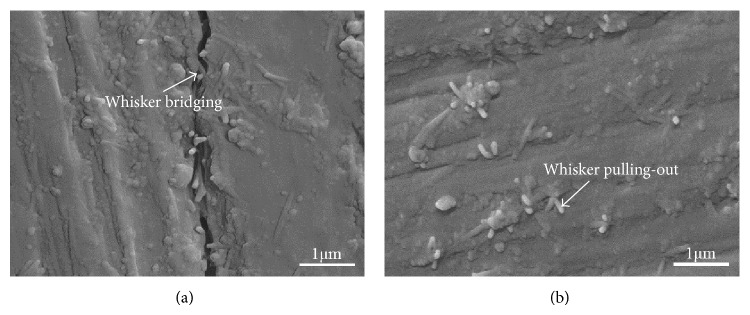
Toughening mechanism of 63s glass/HANw scaffolds: (a) indentation crack propagation, (b) fracture surface.

To better illustrate the reinforcement mechanisms, the micrograph of crack propagation of 63s glass scaffolds with HANw was presented in Figure 5(a). The whisker bridging in the crack propagation path could be observed clearly. When the matrix was subjected to crack-forming stresses, partial debonding of the whiskers along the crack line occurred to form whisker bridging. In other words, the continuous propagates of microcracks would be inhibited so that the interfacial fracture energy was consumed during this process. As the stress and size of the displacement further increased, the whisker pulling-out became the primary reinforcement mechanism. Fracture surface of the scaffolds was presented in Figure 5(b). On the fracture surface, a number of HANw pulled out from the 63s glass matrix. Moreover, the breakage of HANw also occurred. This indicated that HANw had a strong interfacial bonding with the 63s glass so that the fracture did not occur preferentially at the 63s glass/HANw interface.

It was believed that the incorporate effects of whisker bridging and whisker pulling-out absorbed crack propagating energy during fracture, which resulted in the improved fracture toughness. According to the mechanisms research, it could be known that the composite scaffolds with 10 wt.% HANw could obtain desirable results.

### 3.3. Formation of Apatite Layer

**Figure 6:**
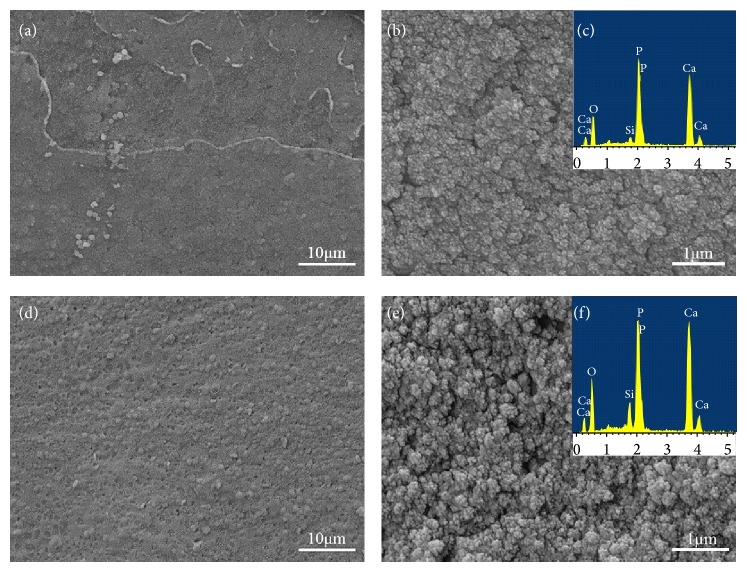
Microstructures of 63s scaffolds with 10 wt.% HANw ((a) low and (b) high magnified image) and 10 wt.% HANp ((d) low and (e) high magnified image) after immersion in SBF for 7 days: (a) low and (b) high magnified image. EDS of the mineral layer ((c) 63s glass/HANw scaffolds; (f) 63s glass/HANp scaffolds).

Surface morphology of composite scaffolds containing 10 wt.% HANw and composite scaffolds containing 10 wt.% HANp soaked in SBF for 7 days was shown in Figure 6. Compared to the scaffolds before SBF (Figure 2), there was a mineral layer deposited on both scaffolds after SBF. At a higher magnification (Figures 6(b) and 6(e)), the mineral layer on both surfaces seemed to be similar composed of a fine structure of nanoparticles with sizes in about 60 nm.

**Figure 7:**
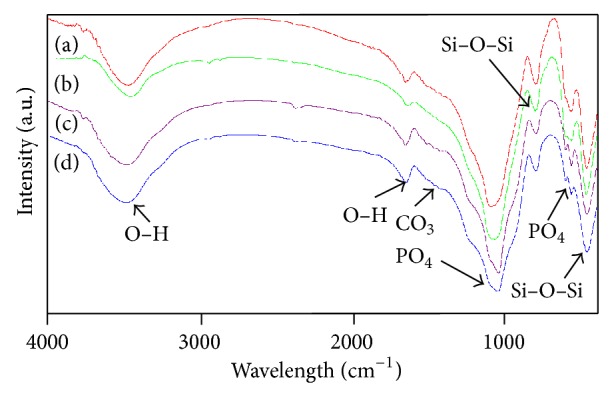
FTIR spectra of scaffolds made of 63s glass with 10 wt.% HANw ((a) before and (c) after being soaked in SBF), 63s glass with 10 wt.% HANp ((b) before and (d) after being soaked in SBF).

EDS analysis (Figures 6(c) and 6(f)) of the mineral layer composition revealed a Ca/P atomic ratio of 1.32 and 1.34, respectively, which were close to the Ca/P atomic ratio of physiological apatite (1.35–1.46) [28]. FTIR spectra of the 63s glass scaffolds with 10 wt.% HANw and 63s glass scaffolds with 10 wt.% HANp surfaces before soaking were shown in Figures 7(a) and 7(b), respectively. The bands at around 3455 and 1647 cm^−1^ are attributed to O–H in adsorbed water [29]. The absorption peaks at 1039 and 601–566 cm^−1^ are assigned to *ν*3 and *ν*4 PO_4_^3–^ group [30, 31], respectively. The bands located at 800 and 464 cm^−1^ are ascribed to silicate group [32]. After soaking in SBF (Figures 7(c) and 7(d)), the intensity of PO_4_^3–^ absorption peak at 566 cm^−1^ was increased. Simultaneously, some carbonate absorption bands at around 1500 cm^−1^ could be recognized [33]. The results indicated that the new HCA layer was precipitating on both surfaces of the composites scaffolds during soaking in SBF.

### 3.4. Cell Morphology

**Figure 8:**
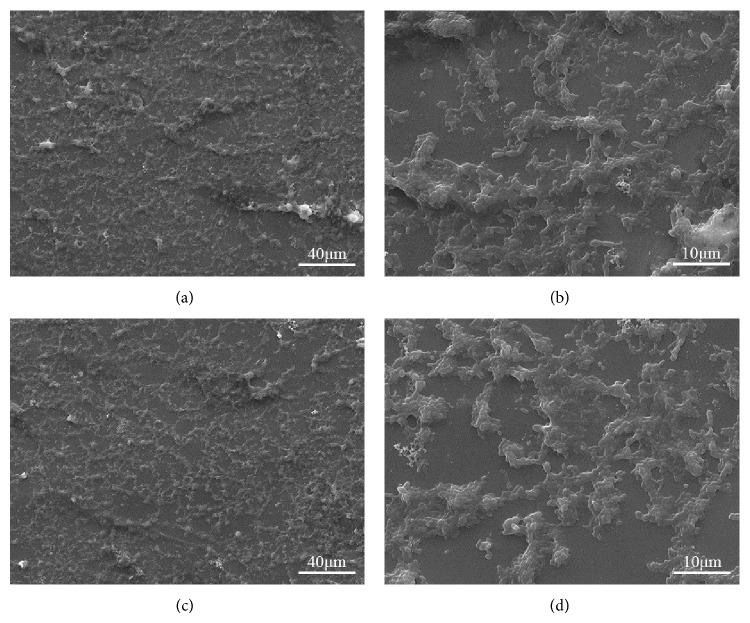
MG-63 cells cultured on 63s scaffolds with 10 wt.% HANw ((a) low and (b) high magnified image) and 10 wt.% HANp ((c) low and (d) high magnified image) for 5 days.

The morphologies of MG-63 cells after 5 days of being cultured on the 63s glass scaffolds with 10 wt.% HANw and 63s glass scaffolds with 10 wt.% HANp were shown in Figure 8. Attached cells on both scaffolds have the similar morphologies with polygonal appearance and numerous pseudopodia. This result indicated that cells can attach and spread well on both surfaces. Cells were also seen to connect to each other by forming extra cellular matrix (ECM), which plays a key role in cell migration and could be observed on both cells and scaffolds. Wide distribution of the ECM and continuous increase in cell aggregation indicated high cell activity on both composites scaffolds.

## 4. Conclusions

The 63s glass/HANw and 63s glass/HANp scaffolds with controllable porous architecture were manufactured by SLS, respectively. 63s glass maintained its amorphous state after laser sintering. Owing to the uniform dispersion of HANw and the strong interface physical bonding between 63s glass and HANw, the compressive strength and fracture toughness increased markedly with increasing HANw content from 0 to 10 wt.%. The incorporate effects of whisker bridging and whisker pulling-out were also observed. The introduction of 10 wt.% HANw to 63s glass scaffolds can produce more effective improvements in mechanical properties than did HANp. Meanwhile, the scaffolds with 10 wt.% HANw doping exhibited favorable apatite-forming bioactivity and excellent cellular biocompatibility. The results of all these studies indicated that HANw reinforced 63s glass scaffolds could be an ideal candidate for bone tissue engineering.
